# Synchronization of Chaotic Systems and Its Application in Security Terminal Sensing Node of Internet of Things

**DOI:** 10.3390/mi13111993

**Published:** 2022-11-17

**Authors:** Yi-You Hou

**Affiliations:** Department of Intelligent Commerce, National Kaohsiung University of Science and Technology, Kaohsiung 824004, Taiwan; yyhou@nkust.edu.tw

**Keywords:** chaotic system, IoT, PID controller, QSMC, security

## Abstract

Recently, with the rapid development of data and information, it has become necessary to establish secure communications and appropriate security services to ensure a secure information exchange process. Therefore, to protect the privacy and confidentiality of private data, in this research, we use the Lorenz chaotic system to generate chaotic signals and apply them to the encryption of the communication of the Internet of Things (IoT) terminal sensor nodes. In addition, we design a simple proportional–integral–derivative (PID) controller and a quasi-sliding mode controller (QSMC) to synchronize the master-slave chaotic systems for decrypting the signals. Then, we encrypt the environmental signals measured from the IoT node at the transmitting side (master) and send them to the receiving side (slave). After the receiving side receives the encrypted signals, it decrypts them with the PID controller. Thus, the security of IoT information can be assured and realized.

## 1. Introduction

The term Internet of Things (IoT) originated in 1999, and was proposed by Kevin Aston of the MIT Auto-ID Center. With the current rapid development of technology, several methods are available for information transmissions, such as Wi-Fi, Bluetooth, and various Internet of Things communication protocols. However, with the advent of these methods, “information security” has become a crucial and inevitable concern. If important information is stolen by others, it is likely to cause irreparable impacts. Furthermore, there has been considerable focus and attention paid to the security of personal information, such as private biomedical information and home information. Thus, the secrecy of personal information must be ensured. Therefore, designing an effective information encryption system is an important goal that this study looks to achieve. Traditional encryption methods can be classified into symmetric encryption (e.g., data encryption standard, DES) and asymmetric encryption (e.g., RSA, ElGamal, and Paillier) [1,2,3]. The basic principle of symmetric encryption is to use Shannon’s concept of multiple encryptions, and apply confusion and diffusion for converting plain text into other formats and spreading every small part of the plain text to each part of the ciphertext to encrypt the information.

Many asymmetric encryption methods have been proposed, such as RSA, ElGamal, and Paillier encryption. These encryption methods mainly use mathematical computation and encrypt important information to avoid its decryption. However, the above algorithms can only be run under the integer domain. In this study, we use signals generated by two chaotic systems to encrypt the IoT signals/information and design a proportional–integral–derivative (PID) controller [4] and a quasi-sliding mode controller (QSMC) [5,6] to synchronize the systems and then recover the IoT signals/information.

In 1989, Ott et al. first proposed a method for controlling chaotic systems and named it the OGY method [7]. Subsequently, Pecora proposed the idea of synchronization control between two independent chaotic systems [8].

A chaotic system is a nonlinear dynamic system with complicated behaviors. Lorenz first used this system in an atmospheric simulation equation in 1963 [9]. However, it did not attract the attention of scientists until 1978. A chaotic system is extremely sensitive to initial conditions [10]. Butterfly effects can be generated by slight changes in the initial conditions, as well as by different attractors. There are various chaotic systems available, including the Hénon map [11], dynamic system in discrete time, Rössler attractor [12,13], and Lorenz oscillator, all of which are ternary nonlinear equations in continuous time.

Due to its complicated behaviors, the chaotic system has been employed in many domains, including communication, biology, mathematics, physics, and chemistry, as well as economics [14]. Thereafter, controlling/synchronizing chaotic systems and their applications became a research focus in the literature [15].

In this study, it is assumed that the collected IoT signals/information are very important signals, and therefore cannot be exposed to unsafe spaces. This study aims to encrypt, decrypt, and safely transmit the IoT signal/information. We use the chaotic system in the master-slave system, which requires a controller to synchronize the chaotic system.

## 2. Research Methods

### 2.1. Generalized Lorenz Chaotic System

The generalized Lorenz chaotic system generates ternary nonlinear equations in continuous time [16]:

(1) $$\begin{array}{c} {\dot{x}}_{1}\left(t\right)=\sigma \left(x_{2}\left(t\right)-x_{1}\left(t\right)\right)\\ {\dot{x}}_{2}\left(t\right)=\gamma x_{1}\left(t\right)-dx_{2}\left(t\right)-x_{1}\left(t\right)x_{3}\left(t\right)\\ {\dot{x}}_{3}\left(t\right)=-bx_{3}\left(t\right)+x_{1}\left(t\right)x_{2}\left(t\right) \end{array}$$

where σ>0, b>0, *c* and *d* are real parameters. The Chen system (2) is a Lorenz-like system (1), with *d* = −*c*, *c* > 0, γ = *c* − *a*.

(2) $$\begin{array}{c} {\dot{x}}_{1}\left(t\right)=a\left(x_{2}\left(t\right)-x_{1}\left(t\right)\right)\\ {\dot{x}}_{2}\left(t\right)=\left(c-a\right)x_{1}\left(t\right)+cx_{2}\left(t\right)-x_{1}\left(t\right)x_{3}\left(t\right)\\ {\dot{x}}_{3}\left(t\right)=-bx_{3}\left(t\right)+x_{1}\left(t\right)x_{2}\left(t\right) \end{array}$$

The system (2) takes {a,b,c}={35, 3, 28} as system parameters, and its dynamic equation can be obtained in continuous time, as shown in (3).

**Figure 1:**
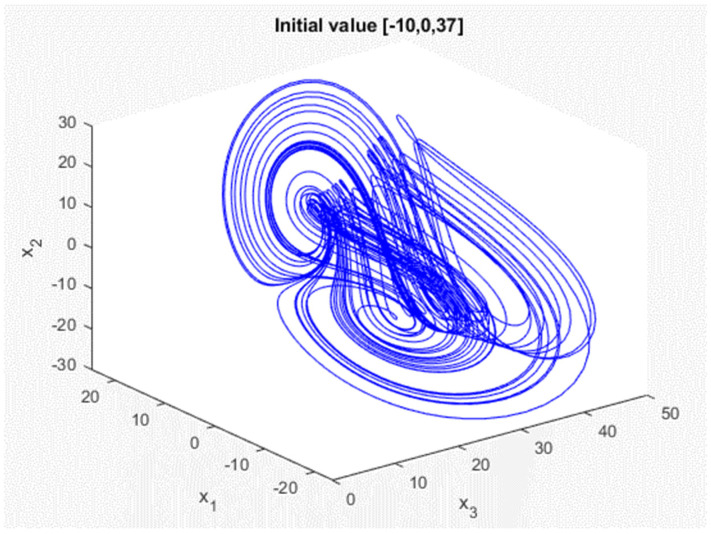
Generalized Lorenz chaotic system response of master.

**Figure 2:**
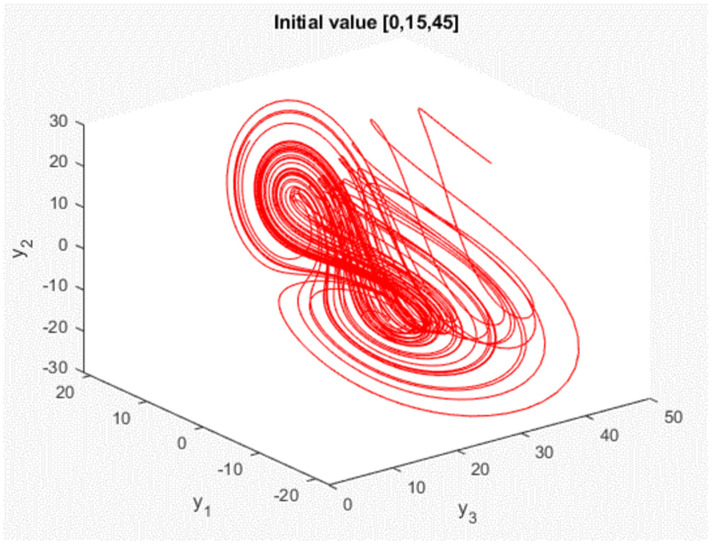
Generalized Lorenz chaotic system response of slave.

In this study, we present the main results for synchronization of chaotic systems (3). We use two chaotic systems: the transmitting side (master) with the state variables $\left[x_{1},x_{2},x_{3}\right]$, and the receiving side (slave) $\left[y_{1},y_{2},y_{3}\right]$, but with different initial conditions of $\left[x_{1}\left(0\right),x_{2}\left(0\right),x_{3}\left(0\right)]=[-10,\;0,\;37\right]$ and $\left[y_{1}\left(0\right),y_{2}\left(0\right),y_{3}\left(0\right)]=[0,\;15,\;45\right]$. Figure 1 and Figure 2 depict the responses of the chaotic system in the master chaotic system and the slave chaotic system in three dimensions with double-scroll attractors, respectively.

(3) $$\begin{array}{c} {\dot{x}}_{1}\left(t\right)=-35x_{1}\left(t\right)+35x_{2}\left(t\right)\\ {\dot{x}}_{2}\left(t\right)=-7x_{1}\left(t\right)+28x_{2}\left(t\right)-x_{1}\left(t\right)x_{3}\left(t\right)\\ {\dot{x}}_{3}\left(t\right)=-3x_{3}\left(t\right)+x_{1}\left(t\right)x_{2}\left(t\right) \end{array}$$

### 2.2. PID Controller Synchronizing Generalized Lorenz Chaotic Systems

Because the IoT signal/information is not continuous, in order to encrypt the IoT signal/information later, we first discretize the system from continuous-time to discrete-time, with a sampling time (*T*) of 0.005 s via MATLAB software; the discrete time system can be obtained as follows (4), where k is the time index [17].

**Figure 3:**
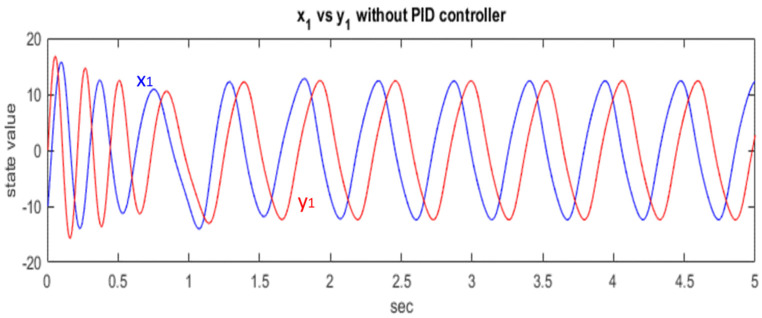
Chaotic systems without PID controller.

In the generalized Lorenz chaotic system, $x_{1}$, $x_{2}$, and $x_{3}$ states affect each other, and thus, we employ the PID controller in one of the states of the chaotic system for synchronization. In this study, we control the first states, $x_{1}$ and $y_{1}$, of the systems. Figure 3 shows the states $x_{1}$ and $y_{1}$ of the master and slave before the application of the PID controller.

Then, we add controller u[k] to the equations in discrete-time at the slave for synchronization, as shown in (4).

(4) $$\begin{array}{c} \begin{array}{ll} y_{1}\left[k+1\right]= & 0.8366y_{1}\left[k\right]+0.1725y_{2}\left[k\right]\\ & -0.000431y_{1}\left[k\right]y_{3}\left[k\right]+u\left[k\right] \end{array}\\ \begin{array}{ll} y_{2}\left[k+1\right]= & -0.0345y_{1}\left[k\right]+1.1471y_{2}\left[k\right]\\ & -0.0053617y_{1}\left[k\right]y_{3}\left[k\right] \end{array}\\ y_{3}\left[k+1\right]=0.9851y_{3}\left[k\right]+0.0049627y_{1}\left[k\right]y_{2}\left[k\right] \end{array}$$

u[k] is the synchronization controller, including the proportional and differential controllers; shown in (5). The proportional controller (*K_p_*) will consider the current error to speed up the time of the transient response so that the chaotic system, slave, will turn into a steady-state and synchronize with master as soon as possible. The integral controller (*K_i_*) will make use of the summation of the past error to eliminate the steady-state’s error. Furthermore, once the proportional and integral controller over controls the system, the overshooting will occur. Here, we are going to use the differential controller (*K_d_*). The differential controller will use the future error to predict the tendency of the system so that it can decrease the rise time and avoid overshooting.

(5) $$\begin{array}{c} u\left[k\right]=K_{p}e\left[k\right]+K_{i}\sum_{i=1}^{k}e\left[i\right]+K_{d}\Delta e\left[k\right]\\ e\left[k\right]=y_{1}\left[k\right]-x_{1}\left[k\right]\\ \Delta e\left[k\right]=e\left[k\right]-e\left[k-1\right] \end{array}$$

**Figure 4:**
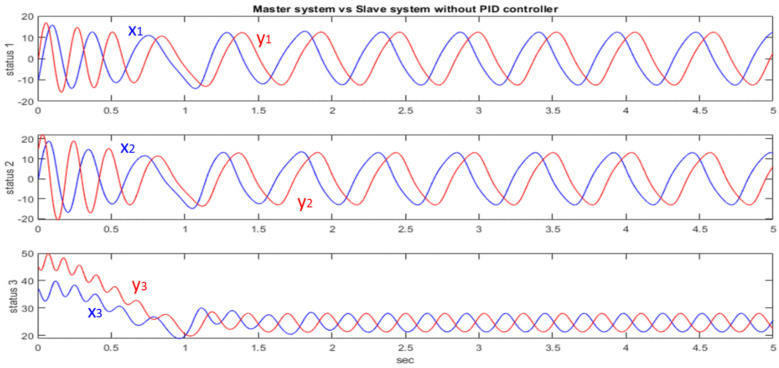
Response to different initial values.

After testing and adjusting various *K_p_*, *K_i_*, *K_d_* values to synchronize two generalized Lorenz chaotic systems with different initial values, we choose the better *K_p_*, *K_i_*, *K_d_* parameters for the subsequent implementation. Finally, we obtain *K_p_* = 0.0025, *K_i_* = 0, *K_d_* = 0.65. To quickly synchronize the two chaotic systems, the *K_i_* value is not used to reduce the occurrence of overshooting. Figure 4 shows the different initial values: $\left[x_{1}\left(0\right),x_{2}\left(0\right),x_{3}\left(0\right)\right]=\left[-10,\;0,\;37\right]$ and $\left[y_{1}\left(0\right),y_{2}\left(0\right),y_{3}\left(0\right)\right]=\left[0,\;15,\;45\right]$ create different system responses. The blue line is the master system side and the red line is the slave system side.

**Figure 5:**
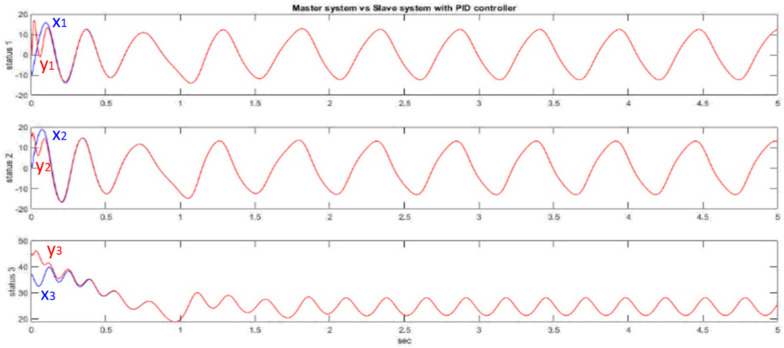
PID controller synchronizing generalized Lorenz chaotic system.

We use the tested PID controller, such as u[k], in (6). Figure 5 shows the effect of the PID controller in synchronizing the two Generalized Lorenz chaotic systems. It can be seen from Figure 5 that the PID controller can quickly synchronize the chaotic system.

(6) u[k]=0.0025e[k]+0.65Δe[k]

### 2.3. Rössler Chaotic System

**Figure 6:**
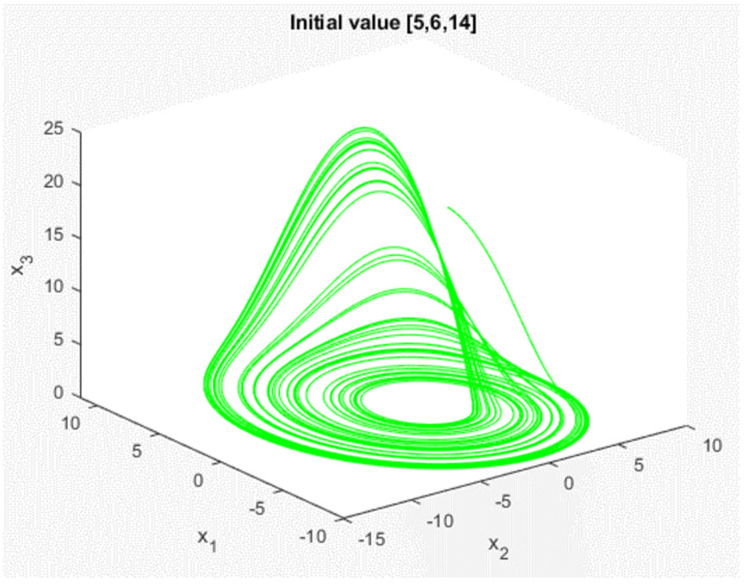
Response of Rössler chaotic system.

In the above, we used the PID controller to synchronize the generalized Lorenz chaotic system. However, this PID controller can only be applied to the master-slave chaotic system in the initial state of our design $\left[x_{1}\left(0\right),x_{2}\left(0\right),x_{3}\left(0\right)\right]=\left[-10,\;0,\;37\right]$ and $\left[y_{1}\left(0\right),y_{2}\left(0\right),y_{3}\left(0\right)\right]=\left[0,\;15,\;45\right]$. If the master and slave chaotic systems have different initial values, the synchronization effect of the above PID controller may not be effective; the chaotic system may not be able to achieve synchronization. Therefore, we want to design a chaotic system where the controller can be applied to any initial value. First, we introduce another chaotic system: the Rössler chaotic system. Its dynamic equation can be obtained in continuous time, as shown in (5), and the dynamic response, as shown in Figure 6, when the initial value is [5, 6, 14].

(7) $$\{\begin{array}{c} {\dot{x}}_{1}\left(t\right)=-x_{2}\left(t\right)-x_{3}\left(t\right)\\ {\dot{x}}_{2}\left(t\right)=x_{1}\left(t\right)+0.2x_{2}\left(t\right)\\ {\dot{x}}_{3}\left(t\right)=0.2-5.7x_{3}\left(t\right)+x_{1}\left(t\right)x_{3}\left(t\right) \end{array}$$

### 2.4. Quasi-Sliding Mode Controller

First, we define the main system as x(t) and the slave system as *y*(*t*), so the error system is e(t)=y(t)−x(t). Because the Rössler chaotic system has a nonlinear term in the third state, the controller is placed in the third state of the error system. The final error system is shown in (8).

(8) $$\{\begin{array}{c} {\dot{e}}_{1}\left(t\right)=-e_{2}\left(t\right)-e_{3}\left(t\right)\\ {\dot{e}}_{2}\left(t\right)=e_{1}\left(t\right)+0.2e_{2}\left(t\right)\\ {\dot{e}}_{3}\left(t\right)=-5.7e_{3}\left(t\right)+y_{1}\left(t\right)y_{3}\left(t\right)-x_{1}\left(t\right)x_{3}\left(t\right)+u\left(t\right) \end{array}$$

In terms of the error system, we hope that the three error states can be as small as possible. We define here that the error system can converge to a very small value. This means that the systems on both sides of the master and servant will reach synchronization.

Next, we need to define a sliding surface. Let the system reach the sliding surface within a limited time, and then move along the sliding surface. In the theoretical description of the sliding mode, the system will be constrained on the sliding surface to reduce the order of the system and eliminate the nonlinear term. Since the nonlinear term is composed of state 1 and state 3, the sliding surface is defined as shown in (9).

(9) $$s\left(t\right)=e_{3}\left(t\right)+\lambda e_{1}\left(t\right)$$

The definition of dynamic error system means that $\delta_{Q}>0$ and $t>t_{Q}$ are entered in the sliding mode control. The solution of any error state of the error system must satisfy $\left|s\left(t\right)\leq \delta_{Q}\right|$ and $t>t_{Q}$. Therefore, when the error system enters the sliding mode, $t>t_{Q}$ and $s\left(t\right)=e_{3}\left(t\right)+\lambda e_{1}\left(t\right)=\delta_{Q}$. Because the error system needs to converge to close to zero for the system to reach synchronization, the value of $\delta_{Q}$ is very small. With this equation s(t), the dynamic equation of the error system can be rewritten as shown in (10).

(10) $$\{\begin{array}{c} {\dot{e}}_{1}\left(t\right)=\lambda e_{1}\left(t\right)-e_{2}\left(t\right)-\delta_{Q}\\ {\dot{e}}_{2}\left(t\right)=e_{1}\left(t\right)+0.2e_{2}\left(t\right)\\ {\dot{e}}_{3}\left(t\right)=\delta_{Q}+\lambda e_{1}\left(t\right) \end{array}$$

After the sliding surface is introduced, the error system is reduced to a second-order system. If we ignore the small value of $\delta_{0}$, it can be expressed as $e_{3}\left(t\right)=\lambda e_{1}$. Now, we just ignore $\delta_{0}$ and consider the response of this second-order system. The second-order system can be simplified as shown in (11).

(11) $$\dot{X}=AX,\;X=\left[\begin{array}{c} e_{1}\left(t\right)\\ e_{2}\left(t\right) \end{array}\right],\;A=\left[\begin{array}{cc} \lambda & -1\\ 1 & 0.2 \end{array}\right]$$

According to the control theory [18], we can know that the transfer function of the pole is $q\left(s\right)=\det(sI-A)=s^{2}-\left(\lambda +0.2\right)s+\left(0.2\lambda +1\right)$. Then, we use Routh-Hurwitz stability [19,20] to find the range of λ, as in (12). Finally, we find −5<λ<−0.2.

(12) $$\begin{array}{cccc} s^{n} & a_{n} & a_{n-2} & a_{n-4}\\ s^{n-1} & a_{n-1} & a_{n-3} & a_{n-5}\\ s^{n-2} & b_{1}=\frac{a_{n-1}a_{n-2}-a_{n}a_{n-3}}{a_{n-1}} & b_{2}=\frac{a_{n-1}a_{n-4}-a_{n}a_{n-5}}{a_{n-1}} & b_{3}\\ s^{2} & 1 & \left(0.2\lambda +1\right) & \\ s^{1} & -\left(\lambda +0.2\right) & 0 & \\ s^{0} & -\left(0.2\lambda +1\right) & 0 & \end{array}$$

So far, we have proved that $\delta_{0}$ must be very small and the range of λ makes state 1 and state 2 of the error system stable. Now, it is necessary to prove that state 3 of the error system can also be stable; to prove that the sliding surface should converge and find the form of the controller. The controller form *u*(*t*) is shown in (13), and the Lyapunov function [21,22] has been used to prove (14); that the sliding surface will converge.

(13) $$u\left(t\right)=-w\eta \left(t\right)\frac{s\left(t\right)}{\left|s\left(t\right)\right|+\delta }$$

(14) $$\begin{array}{c} \delta_{Q}=\frac{w\delta }{w-1}\\ w>1,\;\delta >0\\ \eta \left(t\right)=\left|-\lambda e_{2}\left(t\right)-\left(5.7+\lambda \right)e_{3}\left(t\right)+y_{1}\left(t\right)y_{3}\left(t\right)-x_{1}\left(t\right)x_{3}\left(t\right)\right|\\ v=\frac{1}{2}s^{2}\\ \dot{v}=s\dot{s}\\ ∵\;s=e_{3}+\lambda e_{1}\;∵\;\dot{v}=s\left({\dot{e}}_{3}+\lambda {\dot{e}}_{1}\right)\\ \dot{v}=\eta \left(1-w)(|s|-\frac{w\delta }{w-1}\right) \end{array}$$

**Figure 7:**
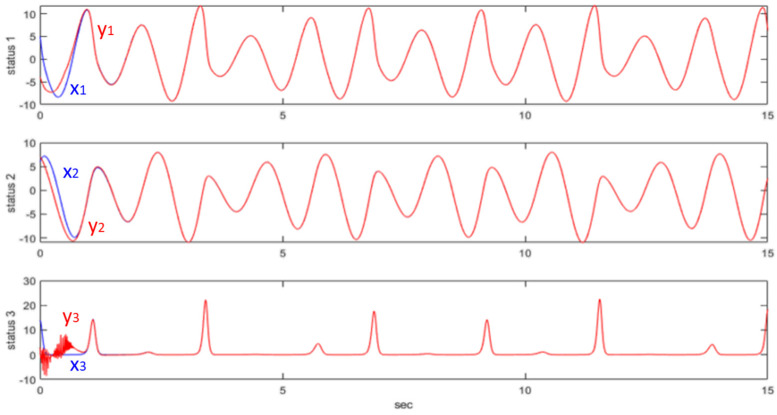
QSM controller synchronizing the Rössler chaotic system.

Therefore, w>1 has been selected from the controller, which means $\dot{v}<0$, when $\left|s\left(t\right)\right|>\delta_{Q}=\frac{w\delta }{w-1}$. This means that |s(t)| will converge to the region of $\left|s\left(t\right)\right|\leq \delta_{Q}=\frac{w\delta }{w-1}$. Then, we conduct a simple simulation: let λ=−1.8, δ=0.03, w=4, and $\delta_{Q}=\frac{w\delta }{w-1}=0.04$. The initial value $\left[x_{1}\left(0\right),x_{2}\left(0\right),x_{3}\left(0\right)\right]=\left[5,\;6,\;14\right]$ and $\left[y_{1}\left(0\right),y_{2}\left(0\right),y_{3}\left(0\right)\right]=\left[-4,\;7,\;3\right]$. The system response is shown in Figure 7.

## 3. Information Security

### 3.1. Chaotic System Encryption Architecture for the Information Security

**Figure 8:**
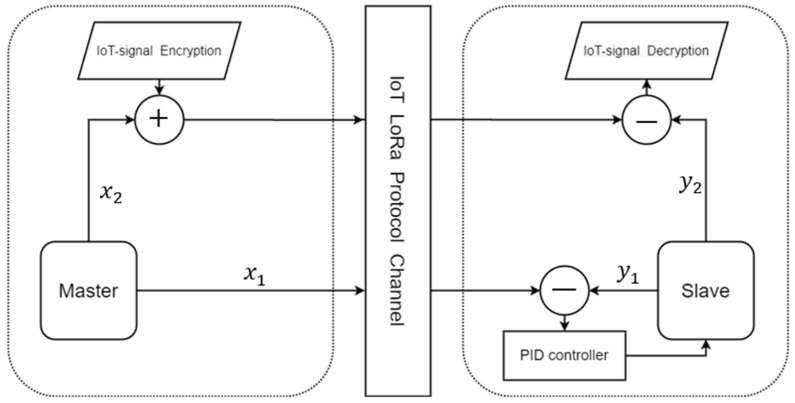
The secure IoT signal system architecture (PID).

In this study, we use the PID controller to synchronize the master-slave chaotic systems, which uses the error to adjust the controller and synchronize the system. Thus, one of the states of the chaotic system must be simultaneously transmitted with the encrypted data. The architecture of the secure IoT system is shown in Figure 8.

In the transmitter side (master system), $x_{2}$ is used in the encryption algorithm via the chaotic masking method, and $x_{1}$ is used for the chaos synchronization by the PID controller design; thus, $x_{1}$ is sent to the slave. When the chaotic system reaches synchronization, we take $y_{2}$ to decrypt the IoT signal. In the middle of the communication system, we use the LoRa module.

### 3.2. Information Security

As can be seen from Figure 8, we use $x_{2}$ of the chaotic system to encrypt the IoT signal. The encryption method is shown in (15).

(15) $$data^{\prime }=data+x_{2}$$

Because the chaotic system has good pseudo-random characteristics, unpredictability of the orbit, sensitivity to the initial state, and control parameters, etc., if a thief steals the encrypted value, they cannot crack it. Even if the thief steals $x_{1}$ of the chaotic system and simulates the response of the transmitting side system, because the chaotic system has a butterfly effect, it is impossible to find $x_{1}$. As long as the system state is worse, the response will be completely different. Finally, the receiving side (slave system) has synchronized the chaotic system $y_{2}=x_{2}$. Therefore, the receiving side can use $y_{2}$ to restore the IoT signal. The decryption method is shown in (16).

(16) $$data=data^{\prime }-x_{2}=data^{\prime }-y_{2}$$

### 3.3. Simulation of Information Security

**Figure 9:**
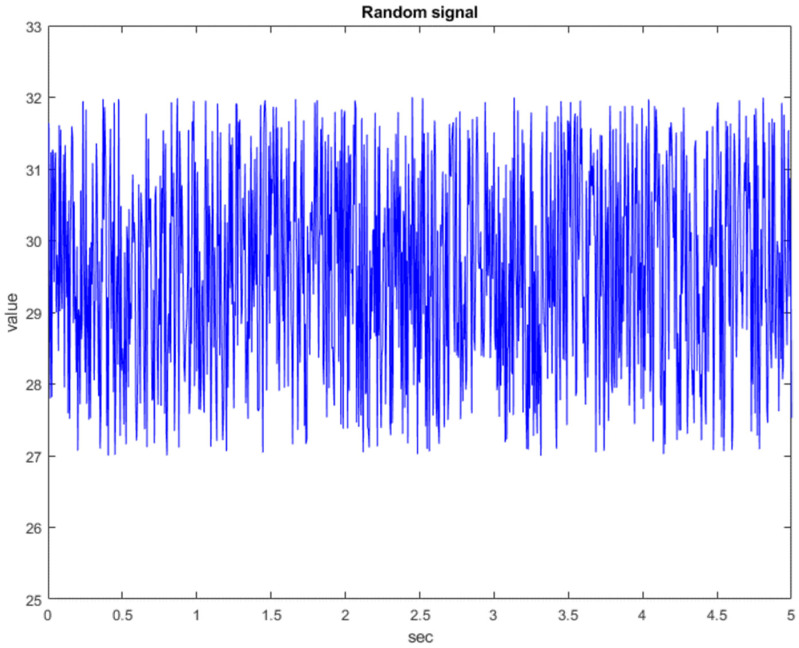
The random signal graph.

Before entering the implementation, we conduct a simulation of information security to test whether this architecture can use a chaotic system to encrypt and decrypt signals. First, we use a random number generator to generate a random signal, as shown in Figure 9.

**Figure 10:**
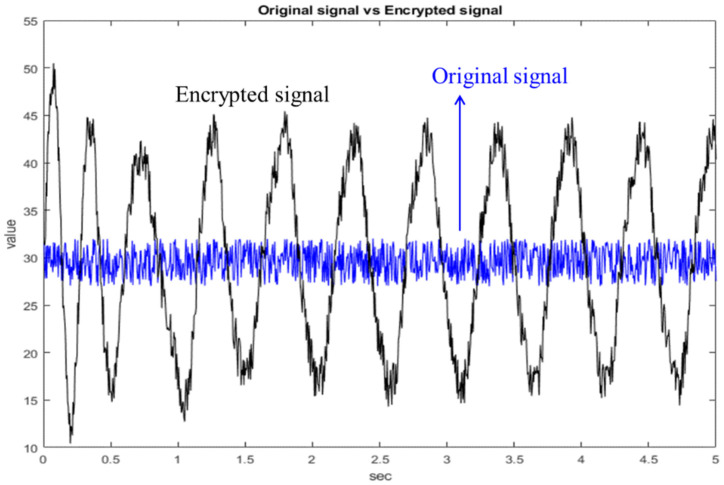
Comparison chart of original signal and encrypted signal.

**Figure 11:**
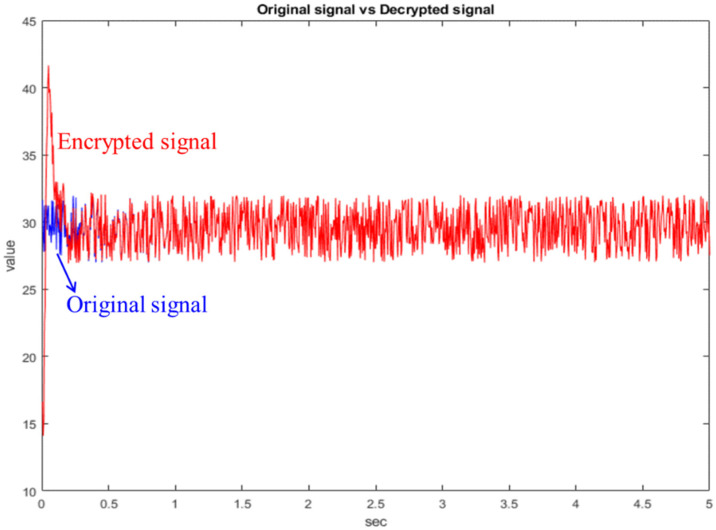
Comparison chart of original signal and decrypted signal.

Next, the random signal is encrypted with the state of the chaotic system, as shown in Figure 10. The blue line is the original random signal and the black line is the encrypted signal. As we can see from Figure 10, the original signal has been completely encrypted. The original random signal cannot be solved from the encrypted signal. Finally, the chaotic system at the receiving side is used for decryption, as shown in Figure 11. As can be seen from Figure 11, the decryption fails before 0.5 s because the chaotic systems at both sides have not reached synchronization. After 0.5 s, the chaotic systems at both sides reach synchronization, so the decryption is successful. Therefore, the simulation proves that this architecture is feasible.

### 3.4. QSMC Synchronized Chaotic System Encryption Architecture for the Information Security

**Figure 12:**
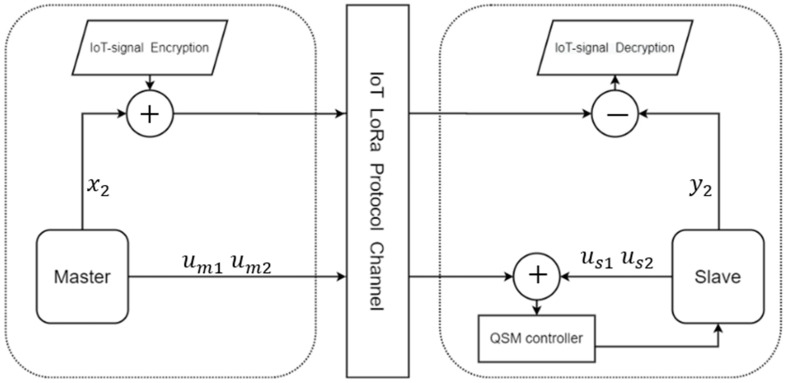
The secure IoT signal system architecture (QSM).

Above, we outlined the encryption architecture using the PID controller. In the same way, we can fit it into the form of QSMC. First, the QSMC can be simplified into a form such as (17). Transmit $u_{m1}\left(t\right)$ and $u_{m2}\left(t\right)$ to the receiving end to realize the sliding mode controller. It is safer than the PID controller, because the value transmitted by the QSMC is a linear combination of the master state and it is not easy to guess the state of the system, and the PID controller has exposed one of the system states. Figure 12 shows the encryption architecture of QSMC.

(17) $$\begin{array}{l} u\left(t\right)=-w\eta \left(t\right)\frac{s\left(t\right)}{\left|s\left(t\right)\right|+\delta }\\ \eta \left(t\right)=\left|-\lambda e_{2}\left(t\right)-\left(5.7+\lambda \right)e_{3}\left(t\right)+y_{1}\left(t\right)y_{3}\left(t\right)-x_{1}\left(t\right)x_{3}\left(t\right)\right|\\ =\left|u_{m1}\left(t\right)+u_{s1}\left(t\right)\right|\\ u_{m1}\left(t\right)=\lambda x_{2}\left(t\right)+\left(5.7+\lambda \right)x_{3}\left(t\right)-x_{1}\left(t\right)x_{3}\left(t\right)\\ u_{s1}\left(t\right)=-\lambda y_{2}\left(t\right)-\left(5.7+\lambda \right)y_{3}\left(t\right)-y_{1}\left(t\right)y_{3}\left(t\right)\\ s\left(t\right)=e_{3}\left(t\right)+\lambda e_{1}\left(t\right)=u_{m2}\left(t\right)+u_{s2}\left(t\right)\\ u_{m2}\left(t\right)=-x_{3}\left(t\right)-\lambda x_{1}\left(t\right)\\ u_{s2}\left(t\right)=-y_{3}\left(t\right)+\lambda y_{1}\left(t\right) \end{array}$$

## 4. Implement

### 4.1. IoT Signal/Information

**Figure 13:**
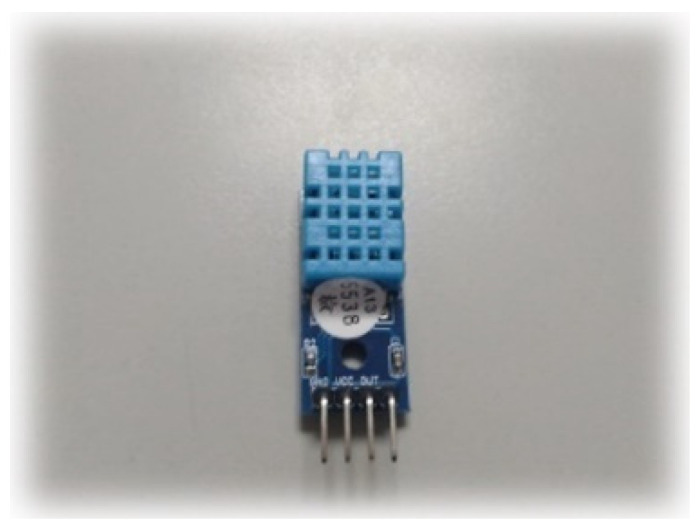
Temperature sensor DHT-22.

**Figure 14:**
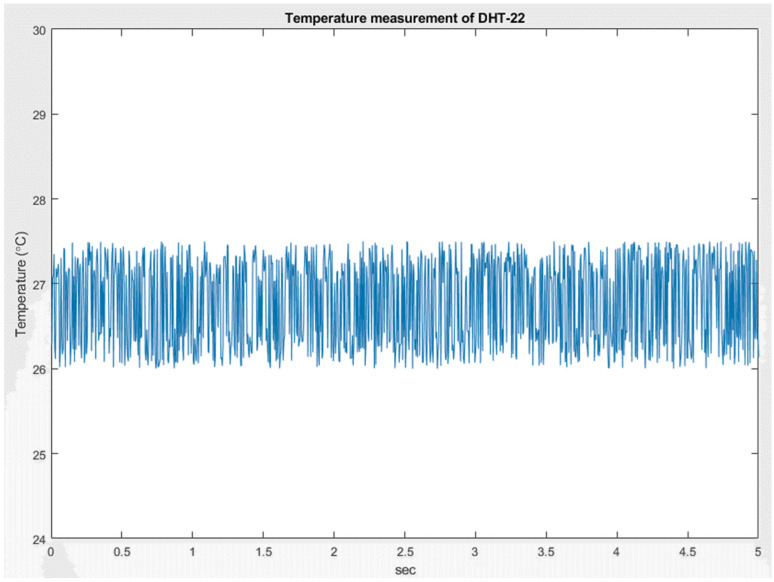
Temperature measured by DHT-22.

In this study, we used the DHT-22 sensor as an example of IoT signal/information, as shown in Figure 13. The DHT-22 sensor is a temperature and humidity composite sensor with a calibrated digital signal output. It uses dedicated digital module acquisition technology, as well as temperature and humidity sensing technology to ensure that the product has extremely high reliability and excellent long-term stability. Therefore, the product has the advantages of excellent quality, ultra-fast response, strong anti-interference ability, and high-cost performance. The temperature and humidity information are the most common signals in the IoT. We used DHT-22 with a sampling time of 0.005 s, as shown in Figure 14.

### 4.2. IoT Communication Channel

**Figure 15:**
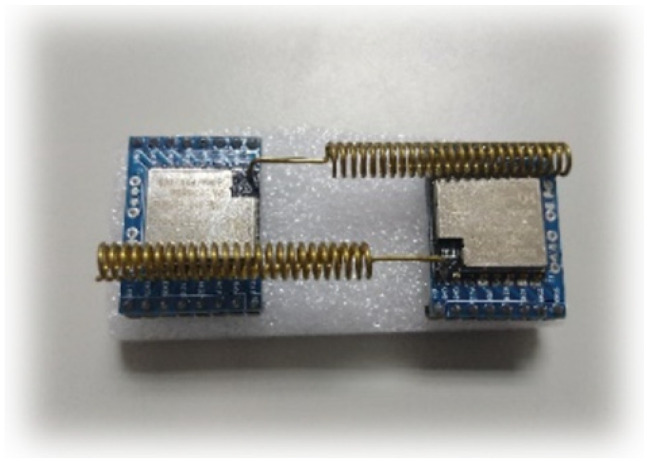
SX LoRa-1278 communication module.

In order to allow temperature sensing data to be transmitted in the IoT. We used the SX LoRa-1278 communication module, as shown in Figure 15. In terms of IoT communication technology, one of the LoRa (Long Range) low-power wide-area network communication technologies was an ultra-long-distance wireless transmission scheme based on spread spectrum technology, adopted and promoted by Semtech. LoRa uses a high spreading factor to obtain a higher signal gain. Compared with the general FSK, the signal-to-noise ratio requires 8 dB, while LoRa only requires −20 dB. This provides users with a simple system that can achieve long-distance, low power consumption, and large capacity, and can then expand the sensor network. Therefore, using the many advantages of LoRa, the nodes of each LoRa module were deployed in the space to collect the required data, such as temperature, humidity, distance, etc. However, because all LoRa frequency bands are publicly shared and free, we needed an encryption system to protect the security of these data.

**Figure 16:**
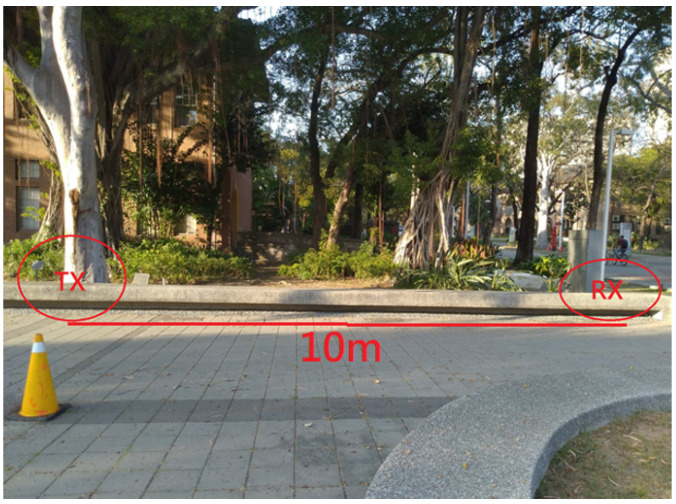
SX LoRa-1278 communication experimental environment.

To ensure the correct rate of the LoRa communication module, we conducted delay time and different distance tests to find a suitable delay time for our indoor applications. We designed an experiment in which the transmitting side transmitted a thousand pieces of data, and the data was generated by the temperature and humidity sensing module, testing the reception rate of the receiving side. Table 1 shows the experimental results. Figure 16 shows the experimental environment. From Table 1, it can be seen that the reception rate of the LoRa module was better at close range, but it was found that the reception rate of the LoRa module was greatly affected when the distance was increased. Therefore, the indoor application of the LoRa module to sense, transmit, and receive various indoor data distances is an important consideration. Finally, we chose a delay time of 200 milliseconds at a receiving rate of 10 m, which was the transmission interval of the LoRa module.

### 4.3. Implementation of the Chaotic Encryption System in IoT Information Security

**Figure 17:**
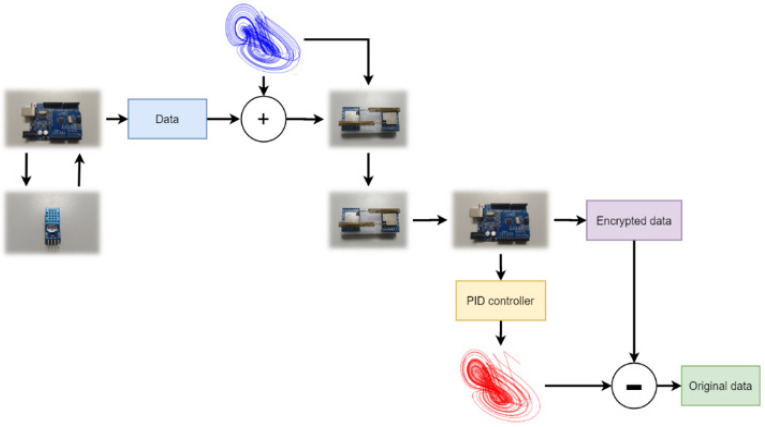
Chaotic system encryption IoT signal architecture diagram.

**Figure 18:**
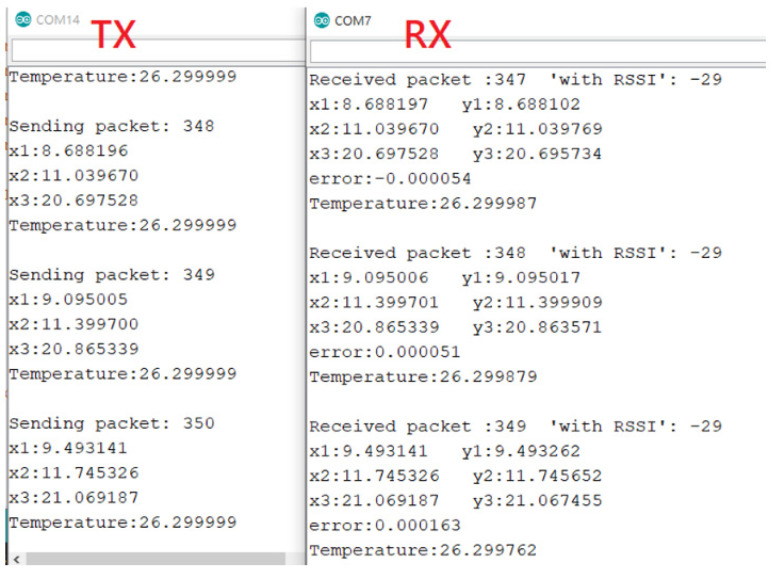
The result of implementing a chaotic encryption system.

First, we used Arduino to connect the DHT-22 to detect temperature values from the environment. The chaotic signal generated by Arduino encrypts the temperature value and then it is transmitted to the receiving side by SX LoRa-1278. The receiving side uses the PID controller to synchronize the chaotic system and then performs decryption. Finally, the temperature value detected by the original DHT-22 is decrypted. The IoT information security system architecture is shown in Figure 17. The results of integrating the LoRa and the chaos system in the ARDUINO interface are shown in Figure 18. As can be seen from Figure 18, the chaotic system reached synchronization when the temperature decryption was successful. The same was true for the encryption and decryption architecture of QSMC.

## 5. Conclusions

The results obtained in this study verify the fact that the characteristics of IoT signals/information after encryption and decryption remain the same, which means that the two chaotic systems are synchronized and generate the same states so that the IoT information remains correct.

We used SX LoRa-1278 to communicate between the two chaotic systems and synchronize them with the proportional–derivative controller or the quasi-sliding mode controller. The experimental results indicate that the master chaotic system successfully transmits the encrypted IoT signals/information to the other side by using the slave chaotic system. Moreover, we obtained the same IoT signals/information after decryption.

Thus, we achieved our goal based on the chaotic system, with a synchronization controller applied to the security of the IoT information.

## Figures and Tables

**Table 1:** SX LoRa-1278 reception rate experiment results.

Distance (m)	Delay Time (ms)	Reception Rate
10	100	95.3%
	125	96.6%
	150	97.4%
	175	100%
	200	100%
20	100	93.3%
	125	94.1%
	150	96.8%
	175	100%
	200	100%
30	100	80%
	125	91.1%
	150	94.4%
	175	100%
	200	100%
40	100	77.2%
	125	81.0%
	150	88.2%
	175	100%
	200	100%

## Data Availability

Data are contained within the article.
